# Delayed APC/C activation extends the first mitosis of mouse embryos

**DOI:** 10.1038/s41598-017-09526-1

**Published:** 2017-08-29

**Authors:** Anna Ajduk, Bernhard Strauss, Jonathon Pines, Magdalena Zernicka-Goetz

**Affiliations:** 1 The Wellcome Trust/Cancer Research UK Gurdon Institute, University of Cambridge, Tennis Court Road, Cambridge, CB2 1QN UK; 2 Department of Embryology, Faculty of Biology, University of Warsaw, Miecznikowa 1, 02-096 Warsaw, Poland; 3 The Institute of Cancer Research, 237 Fulham Road, London, SW3 6JB UK; 4 Department of Physiology, Development and Neuroscience, University of Cambridge, Downing Street, Cambridge, CB2 3GE UK

## Abstract

The correct temporal regulation of mitosis underpins genomic stability because it ensures the alignment of chromosomes on the mitotic spindle that is required for their proper segregation to the two daughter cells. Crucially, sister chromatid separation must be delayed until all the chromosomes have attached to the spindle; this is achieved by the Spindle Assembly Checkpoint (SAC) that inhibits the Anaphase Promoting Complex/Cyclosome (APC/C) ubiquitin ligase. In many species the first embryonic M-phase is significantly prolonged compared to the subsequent divisions, but the reason behind this has remained unclear. Here, we show that the first M-phase in the mouse embryo is significantly extended due to a delay in APC/C activation. Unlike in somatic cells, where the APC/C first targets cyclin A2 for degradation at nuclear envelope breakdown (NEBD), we find that in zygotes cyclin A2 remains stable for a significant period of time after NEBD. Our findings that the SAC prevents cyclin A2 degradation, whereas over-expressed Plk1 stimulates it, support our conclusion that the delay in cyclin A2 degradation is caused by low APC/C activity. As a consequence of delayed APC/C activation cyclin B1 stability in the first mitosis is also prolonged, leading to the unusual length of the first M-phase.

## Introduction

The time between NEBD and the onset of anaphase is one of the most important periods for genomic stability as the cell must ensure that all the chromosomes are attached to the mitotic spindle before sister chromatids separate. This is achieved by regulating the activity of the key ubiquitin-ligase in mitosis, APC/C. In a typical mitotic division, the APC/C is activated at NEBD and one of its first substrates is cyclin A2^1, 2^. Cyclin A2 is required for cells to enter M-phase and it is targeted by the APC/C even though the SAC is active, monitoring unattached chromosomes^3, 4^, and generating the Mitotic Checkpoint Complex (MCC) that inhibits the APC/C. Cyclin A2 can be degraded when the SAC is active because it can compete with the MCC component, BubR1, to bind Cdc20^5^. The cyclin A2-Cdc20 complex then binds to the APC/C through a Cks protein^6, 7^. This mode of recognition allows cyclin A2 to be preferentially ubiquitylated by the APC/C over securin and cyclin B1, when APC/C activity is limited^8^.

By contrast, the timing of cyclin B1 and securin degradation is controlled by the SAC, which prevents the APC/C from recognising cyclin B1 and securin by inactivating Cdc20. One molecule of Cdc20 is incorporated into a complex with the Mad2, BubR1 and Bub3 proteins to form the MCC that itself can inhibit a second molecule of Cdc20^9–13^. Once all the chromosomes have attached correctly to the microtubules of the spindle through their kinetochores, the SAC is inactivated and Cdc20 is released to activate the APC/C against cyclin B1 and securin^14, 15^, leading to the activation of separase and subsequently M-phase exit. The SAC ensures that sister chromatids will segregate to opposite spindle poles once the cohesion complexes are cleaved by separase.

The first mitotic division is highly unique as it is markedly longer than subsequent divisions in many species, including mouse^16, 17^, Xenopus^18, 19^, sea urchins and nematodes^19^. In mouse embryos the first mitosis lasts for 90–120 min, whereas the second lasts only 60–80 min^16, 17^. The human first embryonic mitosis is also very long (approximately 2,5–3hrs^20–24^), and although there are no published data on the length of the second embryonic mitosis, some observations confirm that it tends to be markedly shorter (R. Milewski, J. Czerniecki, S. Wołczyński, unpublished data). By comparison, in somatic cells the length of M-phase varies between 30 and 60 min, depending on the cell type and on the length of the SAC-regulated prometaphase, i.e. the period when the spindle and correct kinetochore-microtubule attachments are formed^25–29^.

One of the mechanisms that might prolong the first embryonic M-phase involves Emi2, an inhibitor of Cdc20^30–32^. The accumulation of Emi2 in metaphase II oocytes inhibits APC/C and thus maintains high levels of cyclin B1 and securin prior to fertilization^33, 34^. Sperm penetration releases the oocyte from the Emi2-induced M-phase arrest by triggering phosphorylation of Emi2 by Ca^2+^/calmodulin dependent kinase II (CaMKII) and Plk1, which subsequently targets it for degradation^30, 35–37^. Emi2 protein reappears in zygotes and it has been hypothesised that this contributes to the prolonged zygotic M-phase^32, 38^. Alternatively, zygotic M-phase has also been proposed to be prolonged by a pool of stable cyclin A2 that inhibits efficient ubiquitination of cyclin B1 and securin by the APC/C^39, 40^.

Here, we have investigated how the APC/C is regulated at the 1- to 2-cell transition in mouse embryos by assaying the degradation of cyclin A2 and cyclin B1. We find that, unlike in somatic cells, the APC/C does not appear to be activated at NEBD because we find cyclin A2 is stable in cells for over 30 min after NEBD, and cyclin B1 is stable for more than 45 min. We show that this delay in APC/C activation is most likely not caused by residual Emi2 but instead depends on Plk1 activity. Moreover, our experiments reveal that cyclin A2 degradation is repressed by the SAC. Thus, we find that the prolonged first mammalian mitosis correlates with an unusual behaviour of the APC/C.

## Results

### Spatiotemporal dynamics of cyclin A2 and cyclin B1 in mouse zygotes

**Figure 1:**
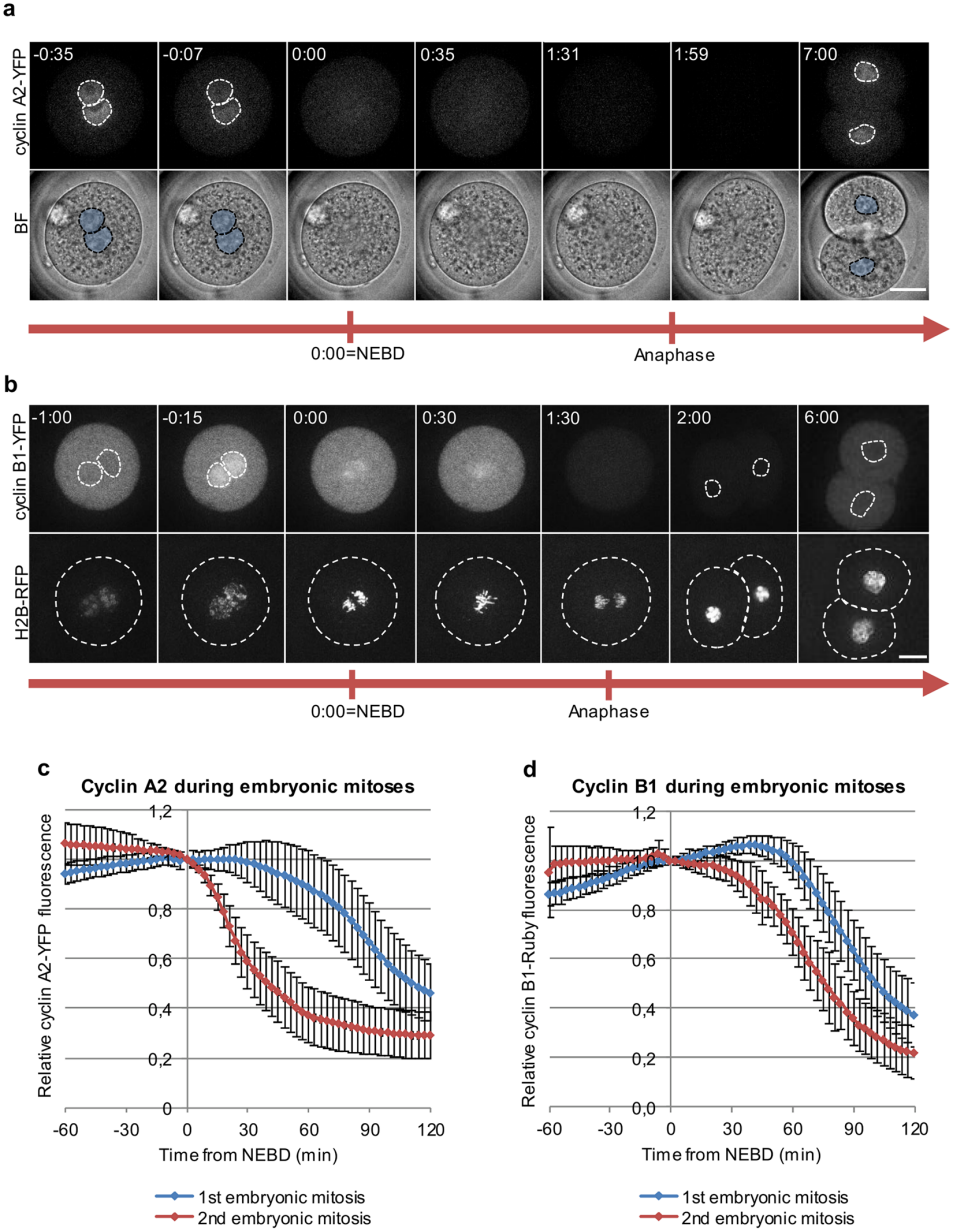
Spatiotemporal dynamics of cyclins A2 and B1 in mouse embryos (**a**) Spatiotemporal dynamics of cyclin A2-YFP in dividing zygotes. Selected images from a time-lapse recording, time shown in hours and minutes counted from the timepoint of nuclear envelope breakdown (NEBD). (**b**) Spatiotemporal dynamics of cyclin B1-YFP in dividing zygotes. Selected images from a time-lapse recording, time shown in hours and minutes counted from the timepoint of NEBD. (**c**) Quantification of cyclin A2-YFP degradation in 1- and 2-cell embryos, averaged from 30 and 37 embryos respectively. (**d**) Quantification of cyclin B1-Ruby degradation in 1- and 2-cell embryos, averaged from 55 and 31 embryos respectively. Scale bars in (**a**) and (**b**) are 20 µm. Plots in (**c**) and (**d**) show mean values + /− standard deviation.

To determine how the very first embryonic M-phase is regulated, we assayed the spatiotemporal dynamics of cyclins A2 and B1. To this end, we injected zygotes or 2-cell embryos with synthetic mRNAs encoding either cyclin A2 tagged with YFP (cyclin A2-YFP), or cyclin B1 tagged with YFP or with Ruby (cyclin B1-YFP and cyclin B1-Ruby) and filmed their development through the first two mitotic divisions. We found that cyclin A2 was localized in zygotic pronuclei during interphase, released into the cytoplasm during NEBD, degraded before the onset of anaphase, then resynthesized and imported into nuclei after the completion of division (Fig. 1a, Supplementary Video S1). In 2-cell embryos cyclin A2 degradation started at NEBD, exactly as in somatic cells^1, 2^, but in zygotes it was significantly delayed (median of 39.0 min post NEBD, p < 0.001) and subsequently proceeded at a slow rate (Fig. 1a,c, Table 1). The observation that cyclin A2 remained stably expressed at a high level for over 30 min after NEBD in zygotes indicated that activation of APC/C was delayed rather than partially activated. In the latter situation, cyclin A2 degradation would be initiated at the NEBD but proceed with slower kinetics.

**Table 1:** Mitotic timings in mouse embryos injected with cyclin A2-YFP.

Experimental conditions		Total no.	Time between NEBD and cyclin A2 degradation (min; median (Q1; Q3))	Time between NEBD and anaphase (min; median (Q1; Q3))
1-cell	control	30	39.0 (30.0; 54.0)	111.0 (99.8; 117.0)
	reversine	31	3.0 (0.0; 19.5)^b^	99.0 (93.0; 108.0)
	wt Plk1 OE	22	15.0 (0.0; 26.3)^a^	108.0 (105.0; 117.0)
	BI2536	38	—	—
2-cell	control	37	0.0 (0.0; 0.0)^b,c,e^	57.0 (51.0; 63.0)^b,d,f^

^a^p < 0.05 comparing to control 1-cell embryos,

^b^p < 0.001 comparing to control 1-cell embryos,

^c^p < 0.05 comparing to reversine-treated 1-cell embryos,

^d^p < 0.001 comparing to reversine-treated 1-cell embryos,

^e^p < 0.05 comparing to 1-cell embryos over-e﻿xpressing wt Plk1,

^f^p < 0.001 comparing to 1-cell embryos over-expressing wt Plk1.

Assaying cyclin B1 showed that it was excluded from the nucleus for most of interphase and imported into the nucleus during prophase, approximately 30 min before NEBD. At NEBD cyclin B1 was released into the cytoplasm and bound the mitotic apparatus (Fig. 1b, Supplementary Video S2). We found that cyclin B1 was degraded at a similar rate during the first and second mitotic divisions (Fig. 1d), but, as with cyclin A2, the onset of cyclin B1 degradation in the first mitotic division was significantly delayed in comparison to the second mitotic division (medians of 48.0 and 18.0 min from NEBD, respectively, p < 0.001, Fig. 1b,d, Table 2). The delay in the degradation of cyclin A2 and cyclin B1 corresponded to the prolonged duration of the whole M-phase in zygotes when compared to 2-cell embryos (medians of 111.0 and 57.0 min, respectively, p < 0.001, for cyclin A2-YFP embryos, and 96.0 and 76.5 min, p < 0.001, for cyclin B1-Ruby embryos, Tables 1 and 2). However, when we tested the correlation between the onset time of cyclin degradation and the duration of M-phase, we found that only the delay in cyclin B1 degradation, and not in cyclin A2 degradation, correlated with the length of M-phase (R = 0.67, p < 0.001). Taken together these results indicate that the prolonged duration of the first mitotic division might be caused by the delayed degradation of cyclin B1.

**Table 2:** Mitotic timings in mouse embryos injected with cyclin B1-Ruby.

Experimental conditions		Total no.	Time between NEBD and cyclin B1 degradation (min; median (Q1; Q3))	Time between NEBD and anaphase (min; median (Q1; Q3))
1-cell	control	55	48.0 (40.5; 52.5)	96.0 (87.0; 105.0)
	reversine	57	39.0 (30.0; 45.0)^b^	87.0 (81.0; 96.0)
	wt Plk1 OE	38	28.5 (24.0; 33.0)^a^	78.0 (75.0; 92.3)^a^
	kd Plk1 OE	11	45.0 (39.0; 51.0)	87.0 (82.5; 99.0)
	BI2536	12	93.0 (83.3; 100.5)^b^	—
	BI2536 + reversine	15	90.0 (87.0; 103.5)^b^	—
	cyclin A2 OE (0.07 µg/µl)	41	48.0 (45.0; 51.0)	115.5 (105.8; 128.3)^a^
	cyclin A2 OE (0.2 µg/µl)	12	54.0 (44.3; 60.8)	117.0 (111.0; 135.0)^e^
	cyclin A2 OE (0.4 µg/µl)	10	54.0 (51.8; 67.5)	—
2-cell	control	31	18.0 (15.0; 24.0)^a,c^	76.5 (69.8; 81.0)^a,d^

^a^p < 0.001 c﻿omparing to control 1-cell embryos,

^b^p < 0.05 comparing to control 1-cell embryos,

^c^p < 0.001 comparing to reversine-treated 1-cell embryos,

^d^p < 0.05 comparing to reversine-treated 1-cell embryos,

^e^values calculated only for embryos that completed cytokinesis (9 out of 12).

### Delayed degradation of cyclin A2 does not affect the timing of cyclin B1 degradation

**Figure 2:**
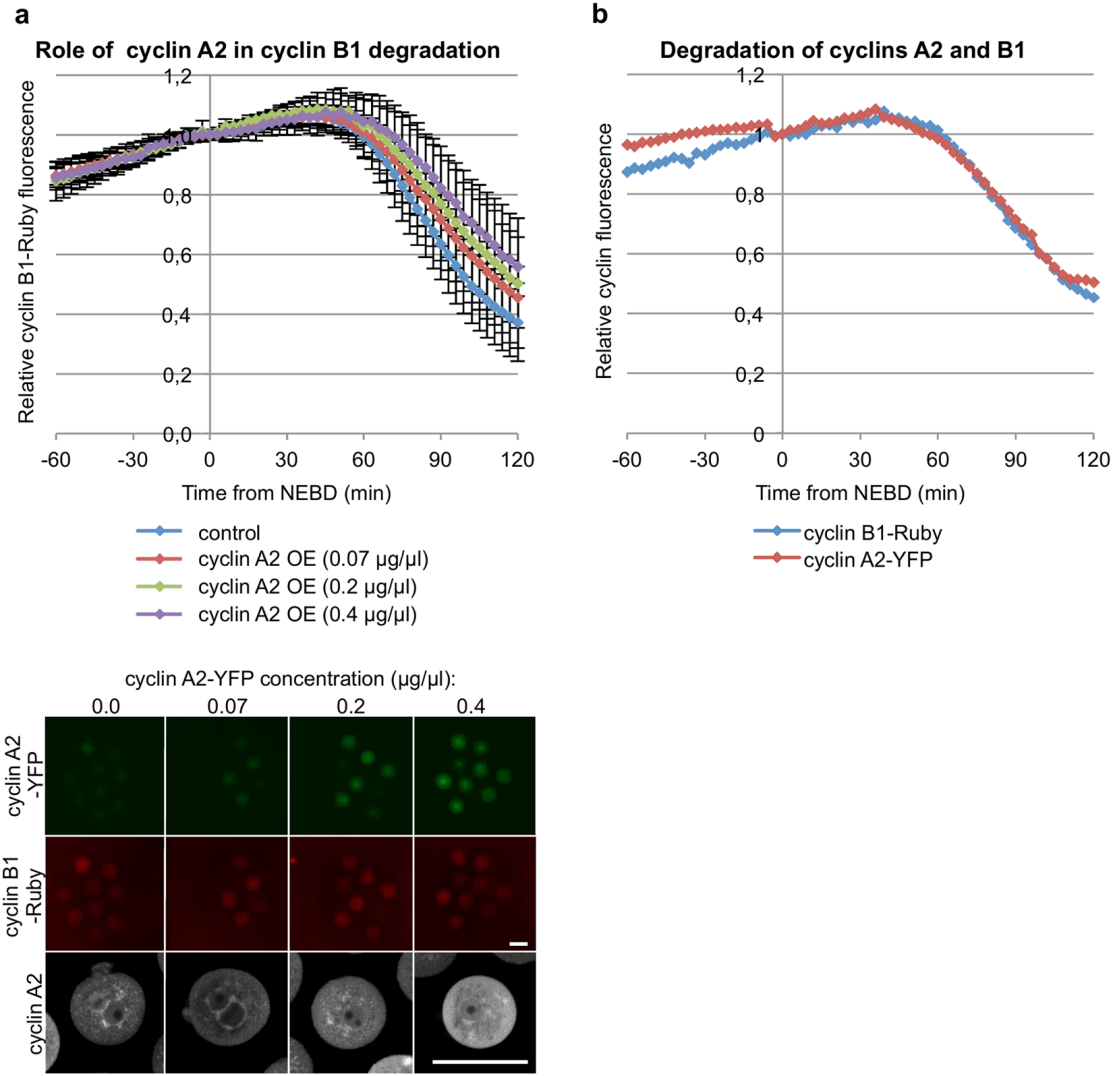
Role of cyclin A2 in regulation of cyclin B1 degradation (**a**) Quantification of cyclin B1-Ruby degradation in zygotes injected with increasing concentrations (0, 0.07 μg/μl, 0.2 μg/μl, 0.4 μg/μl) of cyclin A2-YFP mRNA, averaged from 55, 41, 12 and 10 embryos respectively. Plots show mean values +/− standard deviation. Images show representative zygotes injected with increasing concentrations of cyclin A2-YFP mRNA and the same concentration of cyclin B1 mRNA. Over-expression of cyclin A2 was confirmed by an immunostaining (the bottom panel). Scale bar is 100 μm. (**b**) Quantification of cyclin B1-Ruby and cyclin A2-YFP degradation in a single representative zygote.

As late degradation of cyclin A2 did not seem to correlate with prolongation of the first embryonic M-phase, we directly addressed whether the high levels of cyclin A2 in zygotes after NEBD might reduce the ability of the APC/C to ubiquitylate cyclin B1 and delay its degradation. To test this hypothesis, we examined the effect of over-expressing cyclin A2 on the rate of cyclin B1 degradation. To this end, zygotes were injected with mRNA encoding cyclin B1-Ruby and various concentrations (0.0–0.4 μg/μl) of mRNA encoding cyclin A2-YFP. The increasing concentrations of mRNA translated into increasing amounts of cyclin A2 protein as judged by an increase in fluorescence signal (Fig. 2a). We found that over-expressing cyclin A2-YFP did not change the onset of cyclin B1-Ruby degradation (Fig. 2a, Table 2). We next followed degradation of cyclin B1-Ruby and cyclin A2-YFP simultaneously in zygotes. This revealed that cyclin B1-Ruby started to be degraded on average 3 min after cyclin A2 degradation, and at that time the level of cyclin A2 had only decreased on average by 0.7% (Fig. 2b). These results indicated that the delay in cyclin B1 degradation was not due to the late degradation of cyclin A2.

### The SAC controls both cyclin B1 and cyclin A2 degradation

**Figure 3:**
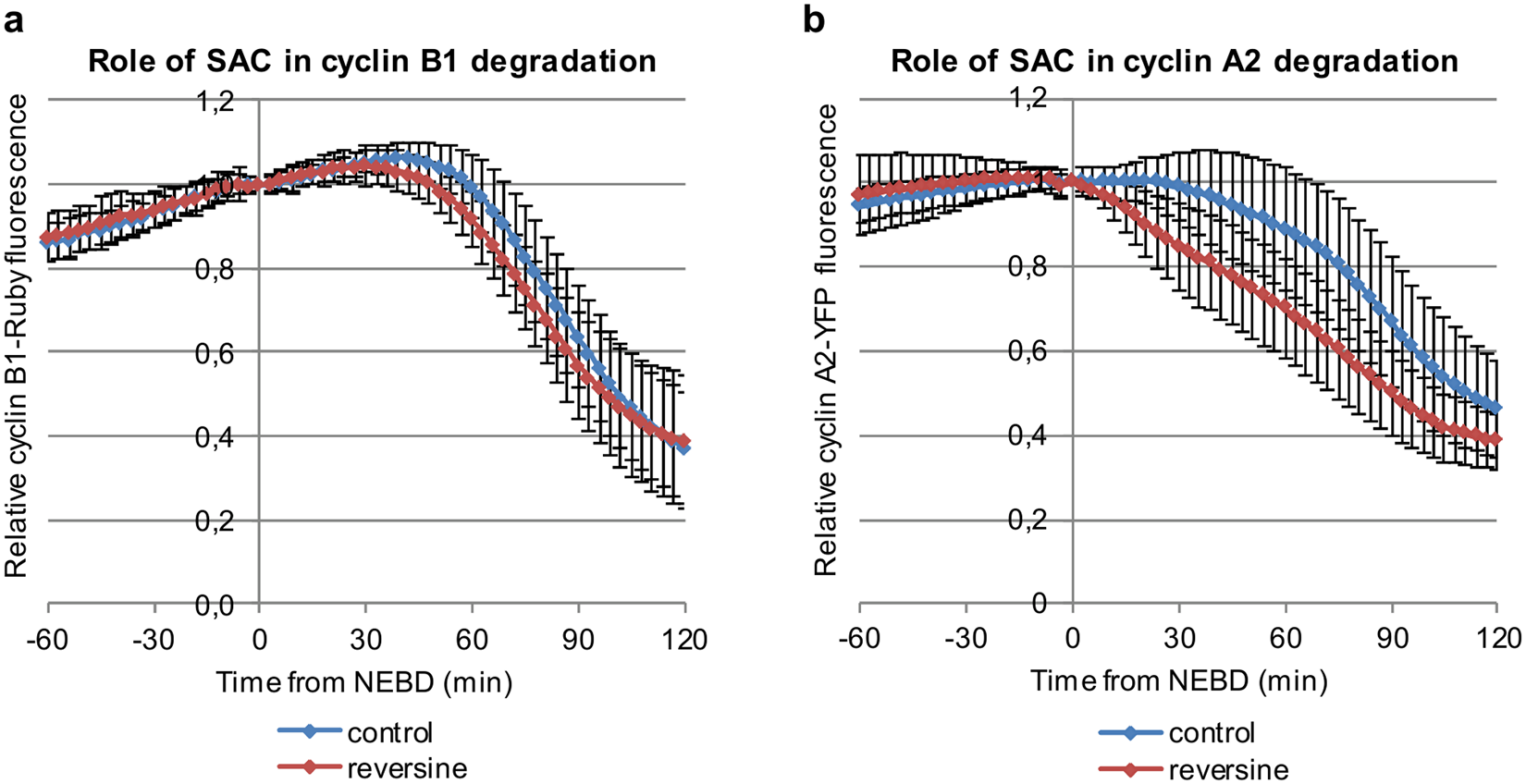
Role of SAC in regulation of cyclin A2 and B1 degradation (**a**) Quantification of cyclin B1-Ruby degradation in control zygotes and zygotes treated with reversine averaged from 55 and 57 embryos respectively. (**b**) Quantification of cyclin A2-YFP degradation in control zygotes and zygotes treated with reversine averaged from 30 and 31 embryos respectively. Plots in (**a**) and (**b**) show mean values +/− standard deviation.

In somatic cells a strong SAC signal does not prevent cyclin A2 degradation, although it slows it down^41^, but it does prevent cyclin B1 degradation^8, 14^. Since degradation of both cyclin A2 and B1 was delayed in the first embryonic M-phase, we wished to investigate whether the SAC was responsible for this. To test this hypothesis, we determined the effect of inhibiting the Mps1 kinase, an essential activator of the SAC^42, 43^, on the dynamics of cyclin A2 and cyclin B1 degradation. We injected zygotes with cyclin A2-YFP or cyclin B1-Ruby and assayed their development in the presence of reversine, an Mps1 inhibitor^42^. We found that inhibiting the SAC shortened the period between NEBD and the onset of cyclin B1-Ruby degradation (to the median of 39 min, p < 0.05, Fig. 3a, Table 2). Similarly, reversine accelerated the onset of securin-GFP degradation (from the median of 36 min to 24 min, p < 0.001; Supplementary Fig. S1a, Supplementary Table S1). Unlike in somatic cells, ablating the SAC in zygotes advanced the timing of cyclin A2-YFP degradation (to the median of 3 min, p < 0.001, Fig. 3b). We concluded that the SAC affects the timing of the cyclin B1 and A2 degradation and thus might be partially responsible for the prolonged first mouse embryonic M-phase. Inactivation of the SAC during the first division did not, however, shorten the time between NEBD and the onset of cyclin B1 or cyclin A2 degradation to the length comparable with the period observed in the subsequent division (p < 0.001 and p < 0.05 respectively, Tables 1 and 2). This indicated that there was an additional mechanism regulating the onset of cyclin B1 and A2 degradation and, in consequence, the length of the first M-phase.

### Emi2 is not involved in regulation of the first embryonic M-phase

**Figure 4:**
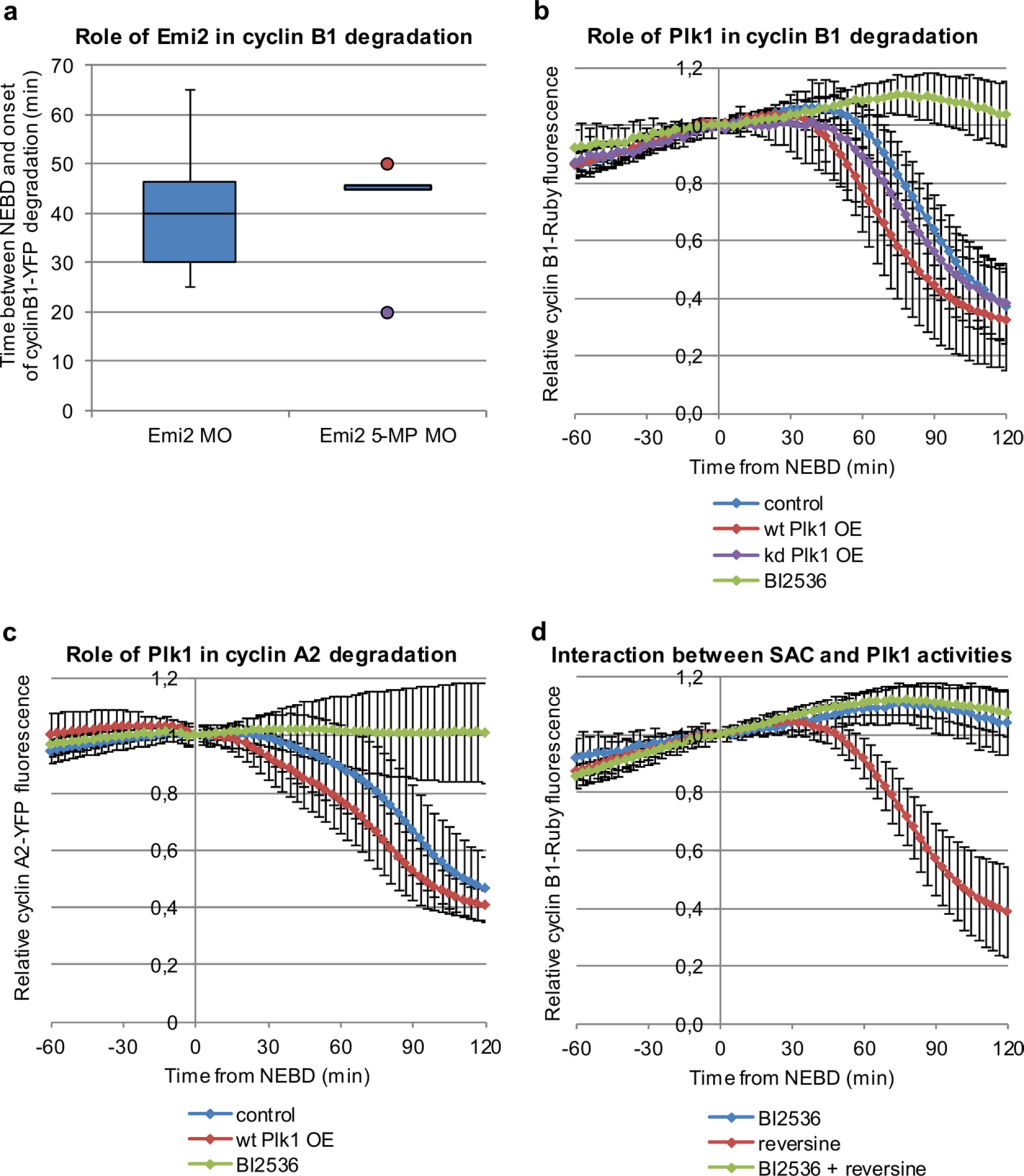
Role of Emi 2 and Plk1 in the regulation of cyclin A2 and B1 degradation (**a**) Time between NEBD and the onset of cyclin B1-YFP degradation in zygotes injected with Emi2 MO and Emi2–5MP MO, averaged from 28 and 11 embryos respectively. The boxes show medians and the first and third quartiles. The whiskers are set at 1.5*IQR above the third and below the first quartile. Outlier values are marked with dots. (**b**) Quantification of cyclin B1-Ruby degradation in control zygotes, zygotes injected with wt Plk1-YFP or kd Plk1-YFP, and zygotes treated with BI2536, a Plk1 inhibitor, averaged from 55, 38, 11 and 12 embryos respectively. (**c**) Quantification of cyclin A2-YFP degradation in control zygotes, zygotes injected with wt Plk1-TagRFP and zygotes treated with BI2536, averaged from 30, 22 and 38 embryos respectively. (**d**) Quantification of cyclin B1-Ruby degradation in control zygotes and zygotes treated with BI2536 or BI2536 and reversine, averaged from 55, 12, and 15 embryos respectively. Plots in (**b**–**d**) show mean values +/− standard deviation.

The first mechanism we considered was APC/C inhibition by maternal Emi2, which is responsible for maintaining oocytes in metaphase II. It had previously been suggested that prolongation of the first embryonic M-phase was caused by the persistence of Emi2 that is resynthesized in zygotes after its degradation triggered by fertilisation^32^. To address whether Emi2 was responsible for the delay in cyclin degradation and the prolonged zygotic M-phase, we depleted Emi2 with specific morpholinos (Emi2 MO), which had been shown to block the translation of Emi2 mRNA^33^. Since Emi2 is required to inhibit the APC/C and maintain oocytes in metaphase II, we assayed the effectiveness of the Emi2 MO to deplete Emi2 by co-injecting Emi2 MO with cyclin B1-YFP into metaphase II oocytes and measured the level of cyclin B1-YFP fluorescence and oocyte activation. We found that 75% of oocytes injected with Emi2 MO (33/44), but only 9% (2/22) oocytes injected with a control MO (Emi2 MO with 5 mismatched pairs of nucleotides, Emi2 5-MP MO) underwent activation within 24 hrs of injection (Supplementary Fig. S2a). Moreover, there was an abrupt decrease in cyclin B1-YFP 12 hrs after microinjection of Emi2 MO, whereas in oocytes co-injected with Emi2 5-MP MO cyclin B1 remained stable (Supplementary Fig. S2b). These results demonstrated that we could efficiently deplete Emi2 through this approach. We injected Emi2 MO into very early zygotes, 2 h post fertilization and well before pronuclei formation, at the time, when Emi2 protein is completely degraded^33^. This ensured that the morpholinos were injected in time to prevent re-synthesis of Emi2 after its degradation at meiotic exit^32, 33^. We found that zygotes injected with Emi2 MO behaved exactly as the control-injected zygotes with no shortening of the M-phase and no difference in the timing of cyclin B1 degradation (medians of 40 and 45 min, respectively, p > 0.05, Fig. 4a).

As a further test of the potential role of Emi2 in prolonging M-phase, we depleted Emi2 by siRNA. To this end, we injected GV stage oocytes with mRNA encoding securin-GFP together with siRNA specific for Emi2 or control siRNA, allowed them to mature *in vitro* until metaphase II, then activated them parthenogenetically and assayed them through the first embryonic division. Emi2 depletion did not accelerate the onset of securin degradation (Supplementary Fig. S3a,b), even though the efficiency of Emi2 siRNA was confirmed by its ability to release metaphase II arrest in injected oocytes (Supplementary Fig. S3c). Taken together, these results show that Emi2 is unlikely to be responsible for the delay in degradation of APC/C substrates during the first embryonic M-phase.

### Plk1 controls both cyclin B1 and cyclin A2 degradation in a SAC-independent manner

We then tested the possibility that Plk1 might be responsible for the delay in cyclin degradation because it is important for the full activation of the APC/C^44-46^. Moreover, Plk1 is essential for the completion of the first embryonic cell cycle in the mouse^47, 48^. To examine whether Plk1 might be involved in regulating cyclin B1 and A2 turnover in the first M-phase in mouse, we injected zygotes with mRNAs encoding wt Plk1 and cyclins (A2 or B1) and then assayed their development. We found that over-expression of wt Plk1-YFP shortened the period between NEBD and the onset of cyclin B1-Ruby degradation to the length typical for the second M-phase (to a median of 28.5 min, p < 0.001 when compared to the first M-phase and p > 0.05 when compared to the second M-phase; Fig. 4b, Table 2). The duration of the whole M-phase was also shortened accordingly (to a median of 78.0 min, p < 0.001 when compared to the first M-phase and p > 0.05 when compared to the second M-phase, Table 2). Over-expression of an inactive, kinase dead Plk1 (kd Plk1-YFP) did not affect the degradation of cyclin B1 (Fig. 4b, Table 2), indicating that the observed acceleration of cyclin B1 degradation required Plk1 activity. Over-expression of wt Plk1-TagRFP also advanced the onset of securin degradation (to a median of 27.0 min, p < 0,05; Supplementary Fig. S1b, Supplementary Table S1). Remarkably, we found that although Plk1 is not required for cyclin A2 to be degraded in somatic cells^49^, over-expressing Plk1-TagRFP significantly accelerated the onset of cyclin A2-YFP degradation in zygotes (to a median of 15 min post NEBD, p < 0.05) (Fig. 4c, Table 1), although it still occurred on average later than in 2-cell embryos (p < 0.05, Table 1).

As a further test of whether Plk1 was involved in regulating the first embryonic M-phase, we inhibited Plk1 with the small molecule inhibitor BI2536^49^ and assessed zygotic division by time-lapse microscopy. This revealed that BI2536 significantly delayed the onset of cyclin B1 degradation (to a median of 93.0 min post NEBD, p < 0.05; Fig. 4b, Table 2). Although we observed some gradual decrease of cyclin B1 in zygotes treated with BI2536, it was never sufficient to induce anaphase (Supplementary Fig. S4a). The inhibition of Plk1 also prevented the degradation of cyclin A2, which persisted at a high level for over 2 h post NEBD (Fig. 4c, Table 1). We found that inhibiting Plk1 activity did not interfere with the formation of a bi-polar spindle, in agreement with a recent report^48^. However, we found that the spindle gradually deteriorated during the prolonged M-phase arrest; specifically the spindle poles became less focused over time and displayed increased numbers of astral microtubules, and in some cases chromosomes were displaced from the metaphase plate (Supplementary Fig. S4a).

To address whether Plk1 altered the length of the first M-phase by altering SAC activity, we asked whether reversine could override the zygotic M-phase arrest induced by Plk1 inhibition. We found that neither reversine, nor injection of mRNA encoding a dominant negative Mad2 (dn Mad2), was able to release zygotes from a BI2536-induced M-phase arrest (Supplementary Fig. S4b). Moreover, reversine was not able to counteract the BI2536-induced delay in cyclin B1 degradation (Fig. 4d, Table 2).

Finally, since our results indicated that Plk1 activity might be limiting in the first M-phase of the mouse embryo, we investigated whether the amount of active Plk1 (i.e. Plk1 phosphorylated at Thr210) differs between M-phase zygotes and 2-cell embryos. Quantitative analysis of the intensity of phospho-Plk1 (pPlk1) immunostaining indicated that active Plk1 was slightly more abundant in M-phase 2-cell embryos than in M-phase zygotes, although the difference did not reach statistical significance (p = 0.058; Supplementary Fig. S5a,b). Interestingly, we observed that there was a subtle, but potentially important, difference in pPlk1 localization in 1- and 2-cell stage embryos. In both the first and the second M-phase pPlk1 localized to the spindle poles and chromosomes; however, in 2-cell embryos it strongly accumulated on kinetochores, whereas in zygotes its chromosomal distribution was more diffuse (Supplementary Figure S5c).

Taken together these results indicate that the degradation of cyclins B1 and A2, and in consequence the prolonged first M-phase, is regulated by a Plk1-dependent mechanism. We propose that a difference in the localization of active Plk1 might potentially be involved in prolongation of the first embryonic M-phase in the mouse.

## Discussion

The first embryonic mitosis in a number of species is strikingly longer than the subsequent embryonic mitoses and mitosis in somatic cells^19, 50^. Indeed, the first zygotic M-phase is unique because during this period two sets of chromosomes, enclosed in separate nuclei, need to form a single metaphase plate. This process is more complicated than its equivalent in a typical mononuclear somatic cell, and therefore most likely more time is required to ensure appropriate chromosome-spindle connections. It appears plausible to us that this is the biological purpose of the prolonged first zygotic M-phase, and particularly the period between NEBD and cyclin B1 degradation.

Here, we have uncovered atypical APC/C behaviour in the first mitotic division that appears to be responsible for this extended M-phase. Our analysis of the spatiotemporal dynamics of cyclin A2 and cyclin B1 indicates that length of the first M-phase is likely a consequence of delayed APC/C activation. We find that cyclin A2 starts to be degraded in zygotes over 30 min after NEBD, in striking contrast to 2-cell embryos or somatic cells where cyclin A2 begins to be degraded at NEBD. The degradation of cyclin B1 and securin in zygotes is also significantly delayed in comparison to mitosis in 2-cell embryos, and to somatic cells^8, 14^. Although it has been hypothesized that the stable pool of cyclin A2 is responsible for the prolonged M-phase in zygotes^39, 40^, our results do not support this idea because we find that cyclin B1 starts to be degraded when the levels of cyclin A2 are still very high. Furthermore, over-expressing cyclin A2 does not delay the onset of cyclin B1 degradation. However, in accordance with published data^1, 2, 51^ we observed that over-expression of cyclin A2 disturbs progression of anaphase and cytokinesis.

Our experiments provide evidence that the SAC regulates APC/C activity towards cyclin A2 in the first M-phase in mouse because inhibiting the SAC both advances and accelerates the degradation of cyclin A2 (and B1). However, our results also indicate that SAC activity is not solely responsible for the prolongation of the zygotic M-phase since inhibiting the SAC accelerated the onset of cyclin degradation by only approximately 10–20 min. This agrees with earlier work showing that SAC proteins, such as Mad2 leave, the kinetochores in zygotes within approximately 20 min of NEBD^17^, and a similar delay in cyclin B1 degradation is caused by the SAC in somatic cells^27, 29^. Thus, there must be another mechanism involved in the delay in degradation of cyclins A2 and B1, and as a result, the prolongation of zygotic M-phase.

Our evidence implicates Plk1 as the additional factor responsible for the first mitotic delay. Plk1 activity appears to be a limiting factor because over-expression of Plk1 accelerates the onset of cyclin A2 and B1 degradation, even though in somatic cells Plk1 activity is not required for the APC/C to target cyclin A2 at NEBD^49, 52^. Moreover, Plk1 over-expression shortens the period between NEBD and the onset of cyclin B1-Ruby degradation to the length typical for the second embryonic mitosis. We show that Plk1 does not act exclusively through the SAC, since ablation of the SAC does not rescue the delay caused by Plk1 inhibition. Plk1 is also unlikely to act through Emi2 since the injection of Emi2-specific morpholinos or siRNAs did not accelerate cyclin B1 or securin degradation during the first embryonic division. However, we cannot completely exclude the possibility that in zygotic M-phase even a very small amount of Emi2 that potentially may remain in the cell after MO- or siRNA-induced depletion is sufficient to inhibit APC/C activity. Indeed, the role of Emi2 in the first mitotic divisions is currently debated in Xenopus embryos^34, 53^. Instead, our evidence would be congruent with a role for Plk1 in activating the APC/C in embryonic divisions through direct phosphorylation of the APC/C complex^45, 46, 52, 54^.

If Plk1 is indeed a limiting factor in the zygotic M-phase, then it should either be expressed at lower levels in zygotes, or be differently regulated. Analysis of transcriptomic data available in the DBTMEE database (RNA-seq Ver2_FPKM collection, http://dbtmee.hgc.jp/)^55^ indicates that Plk1 mRNA expression is higher in zygotes than in 2-cell embryos, but we find that the amount of active Plk1 is comparable – with a tendency to be lower - between M-phase zygotes and 2-cell embryos. Comparing the expression of Plk1 activators / inhibitors also does not reveal a clear difference between these two stages: some transcripts are more abundant in zygotes, others in 2-cell embryos. Alternatively, the difference between the mode of Plk1 action in the first and the second embryonic divisions may relate to the distribution of active Plk1: in contrast to 2-cell embryos, in zygotes, pPlk1 does not accumulate very effectively on kinetochores.

Taken together, we conclude that the APC/C has reduced activity in the first embryonic division that extends the first M-phase. As a consequence we find that cyclin A2 degradation in the first mitosis is atypical, resembling the way in which cyclin B1 is controlled in somatic cells, because its degradation is regulated by both SAC and Plk1 activities. Our observations shed new light on the molecular machinery controlling the unusual length of the first embryonic division.

## Methods

### Embryo collection and culture

All experimental procedures applied to animals were approved by the Home Office (UK) or by the Local Ethical Committe no. 1 (Warsaw, Poland), and were performed in compliance with the national regulations. Mouse F1 (C57Bl6 x CBA) females were superovulated by an intraperitoneal injection of 10 IU of pregnant mare serum gonadotrophin (PMSG, Intervet) followed 48 hrs later by 10 IU of human chorionic gonadotrophin (hCG, Intervet) and mating with F1 males. Zygotes and 2-cell embryos were recovered from oviducts into M2 medium 16–20 hrs and 44 hrs after hCG injection, respectively. MII oocytes were recovered 15 hrs post hCG injection from oviducts of unmated females into M2 medium. GV oocytes were recovered from ovaries 48 hrs after the PMSG injection into M2 medium supplemented with 150 μg/ml dibutyryl cyclic AMP (dbcAMP, Sigma-Aldrich).

### Microinjections and live imaging of embryos

Constructs encoding cyclin B1 tagged with YFP or Ruby (cyclin B1-YFP, cyclin B1-Ruby), cyclin A2 tagged with YFP (cyclin A2-YFP), Plk1 tagged with YFP or TagRFP (wt Plk1-YFP, wt Plk1-TagRFP), kinase dead version of Plk1 tagged with YFP (kd Plk1-YFP), dominant negative version of Mad2 (dn Mad2) and securin tagged with GFP (securin-GFP) were cloned into pBluescript RN3P vector and mRNA was synthesized from the T3 promotor using mMessage mMachine T3 kit (Ambion), as described previously^56^. A construct encoding histone 2B tagged with RFP (H2B-RFP) was cloned into pGEMHE vector and synthesized from the T7 promotor using mMessage mMachine T7 kit (Ambion). mRNAs (needle concentration 0.025 μg/μl for H2B-RFP, 0.07 μg/μl for cyclin B1-Ruby, 0.07 μg/μl for cyclin A2-YFP, 0.2 μg/μl for cyclin B1-YFP, wt Plk1-YFP and kd Plk1-YFP, 0.35 μg/μl for securin-GFP, 0.7 μg/μl for Plk1-TagRFP, 0.8 μg/μl dn Mad2) were injected into a zygote or one blastomere of a 2-cell stage embryo and injected embryos were subsequently cultured in KSOM (Speciality Media, Millipore) for approximately 6 hrs to allow for sufficient protein expression. In some experiments oocytes or embryos were injected with 25 μM siRNA (Sigma-Aldrich) or 2 mM morpholino^33^ (Gene Tools LLC) specific to Emi2 and with appropriate controls (Supplementary Table S2). Emi2 MO was injected into early zygotes (recovered 16 hrs post hCG) together with cyclin B1-YFP, which then were cultured for approximately 8 hrs in KSOM. Emi2 siRNA was injected with securin-GFP into GV oocytes that were afterwards allowed to mature for 16 hrs and subsequently were activated parthenogenetically by incubation in 8% ethanol for 8 min and cultured for 8 hrs in KSOM. Zygotes, parthenogenotes or 2-cell embryos were transferred into M2 medium and imaged in 11–13 planes (5–6 µm apart) every 3–15 min for 12 hrs over the transition from 1- to 2-cell stage or 2- to 4-cell stage. In some experiments M2 was supplemented with 500 nM reversine (Sigma-Aldrich) or 100 nM BI2536 (Axon Medchem). Imaging was performed on a 3i Intelligent Imaging Solutions spinning-disc confocal microscope, Zeiss Axiovert fluorescence microscope or Deltavision fluorescence microscope, all equipped with environmental chambers maintaining temperature of 37.5 °C.

### Immunostaining

Embryos were fixed in 4% PFA (30 min, RT), permeabilised with 0.5% Triton-X100 (30 min, RT) and blocked with 3% BSA. Plk1 phosphorylated at Thr210 was labeled with a polyclonal rabbit antibody (ThermoFisher Scientific; dilution 1:100 in 3% BSA, overnight, 4 °C) and cyclinA2 - with a polyclonal rabbit antibody (Santa Cruz; dilution 1:50 in 3% BSA, overnight, 4 °C), both followed by a secondary anti-rabbit antibody conjugated with Alexa 633 (Molecular Probes, ThermoFisher Scientific; dilution 1:200 in 3% BSA, 1.5 h, RT). Microtubules were stained with mouse anti-tubulin β antibody labeled with FITC (Sigma-Aldrich; dilution 1:50 in 3% BSA, 1.5 h, RT or overnight, 4 °C). DNA was stained with Hoechst 33342 dye (Molecular Probes, ThermoFisher Scientific; 100ng/µl in PBS, 30 min, RT or overnight, 4°C), propidium iodide (Sigma-Aldrich; 3 μg/ml in PBS, 30 min, RT) or chromomycin A3 (Sigma-Aldrich; 10 μg/ml, 30 min, RT or overnight, 4°C). In case of phospho-Plk1 detection, a PhosStop solution (Roche) was added to all stages of immunostaining to inhibit phosphatases.

### Statistical analysis

In order to quantify cyclin B1 or cyclin A2 levels in embryos, projections (sums of all slices) were prepared for all imaged embryos and mean fluorescence intensity was measured for each projection over the time of recording. The intensity values were then standardized with the fluorescence intensity at the NEBD. Polynomial curves were fitted to the data and the onset of cyclin or securin degradation was established as the first time-point after NEBD when the fluorescence decrease between two subsequent measurements was at least 0.01. Statistical analyses of results were performed using Student’s t-test, chi-squared test, Spearman’s rank-order correlation and Kruskal-Wallis test with a post-hoc multiple comparison of mean ranks test.

## Electronic supplementary material

Supplementary Information

Supplementary Video S1

Supplementary Video S2
