# Supplementary material for: Delayed APC/C activation extends the first mitosis of mouse embryos
Source: Sci Rep. 2017 Aug 29;7:9682. doi: 10.1038/s41598-017-09526-1 (PMC5575289; doi:10.1038/s41598-017-09526-1)
Supplement: Supplementary file 1 — Supplementary Information [file 41598_2017_9526_MOESM1_ESM.pdf]

Supplementary Information
for

Delayed APC/C activation extends the first mitosis of mouse embryos

Anna Ajduk, Bernhard Strauss,
Jonathon Pines, Magdalena Zernicka-Goetz

Supplementary Video legends

Supplementary Video S1
Spatiotemporal dynamics of cyclin A2-YFP at 1- to 2-cell transition
Time lapse recording of a representative zygote injected with cyclinA2-YFP mRNA dividing into 2-cell embryo. Video consists of single plane images of YFP and bright field channels. Time shown in hours and minutes, counted from the timepoint of nuclear envelope breakdown (NEBD).

Supplementary Video S2
Spatiotemporal dynamics of cyclin B1-YFP at 1- to 2-cell transition
Time lapse recording of a representative zygote injected with cyclinB1-YFP and histone H2B-RFP mRNAs dividing into 2-cell embryo. Video consists of single plane images of RFP and YFP channels. Time shown in hours and minutes, counted from the timepoint of nuclear envelope breakdown (NEBD).

Supplementary Table S1
Mitotic timings in mouse embryos injected with securin-GFP

	Experimental conditions	Total no.	Time between NEBD and securin degradation (min; median (Q1;Q3))	Time between NEBD and anaphase (min; median (Q1;Q3))
1-cell	control	19	36.0 (30.0 ; 40.5)	90.0 (84.0 ; 93.0)
	reversine	6	24.0 (21.8 ; 28.5)[b]	73.5 (72.0 ; 77.3)[a]
	wt Plk1 OE	19	27.0 (24.0 ; 28.5)[b]	75.0 (72.0 ; 81.0)[a]

[a] $p<0.001$, [b] $p<0.05$ comparing to control embryos

Supplementary Table S2
Morpholinos and siRNAs used in the experiments

Type of morpholino:	Sequence 5'to 3':
Emi2 MO	ATT GCT TCC TGC TCT GTG GCT GGC T
Emi2-5MP (with 5 mismatched pairs of nucleotides) MO	ATT CCT TGC TGC TGT GTG CCT GCC T

Type of siRNA:	Sequence 5' to 3':
Emi2 siRNA	GUC AAA UAA UGA AGU CUG U [dT][dT]<br>ACA GAC UUC AUU AUU UGA C [dT][dT]
control siRNA	MISSION siRNA Universal Negative Control 1 (Sigma-Aldrich)

Supplementary Figure S1

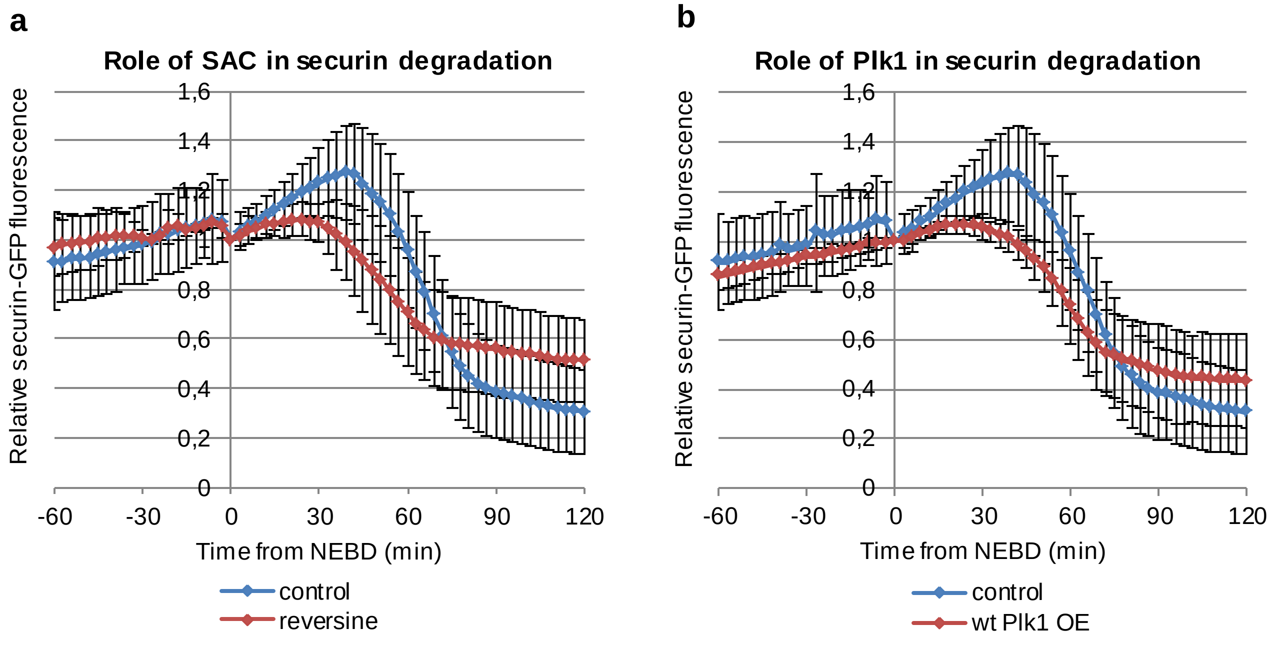

Supplementary Figure S1
SAC- and Plk1-dependent regulation of securin degradation in zygotes
(**a**) Quantification of securin-GFP degradation in control zygotes and zygotes treated with reversine, averaged from 19 and 6 embryos respectively. (**b**) Quantification of securin-GFP degradation in control zygotes and zygotes injected with wt Plk1-TagRFP, averaged from 19 and 19 embryos respectively. Plots (**a**) and (**b**) show mean values +/- standard deviation.

Supplementary Figure S2

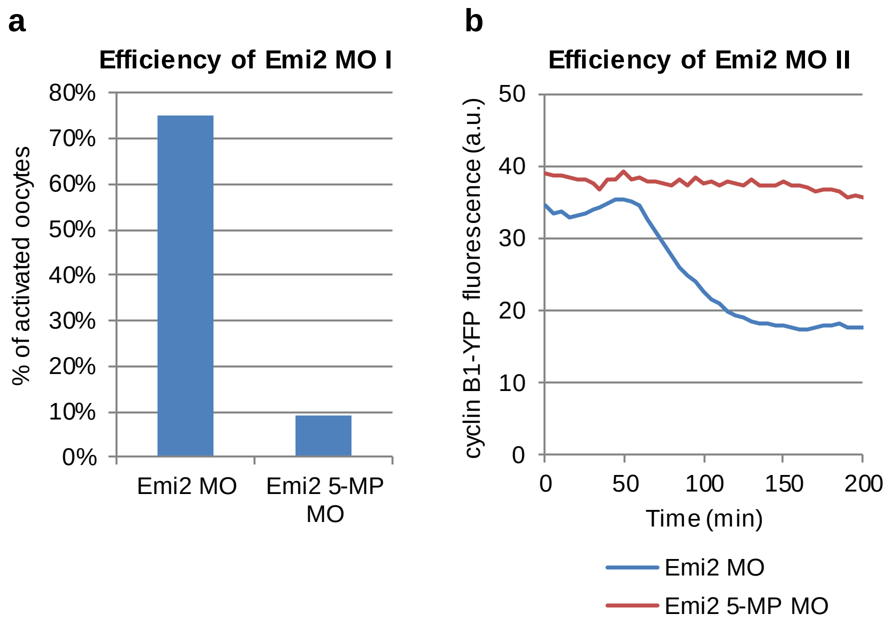

Supplementary Figure S2

MO-mediated Emi2 depletion in oocytes

(**a**) Percentage of oocytes activated after injection with Emi2 MO or Emi2-5MP MO (n=36 and 21, respectively). (**b**) Cyclin B1-YFP levels in representative oocytes injected with Emi2 MO or Emi2-5MP MO.

Supplementary Figure S3

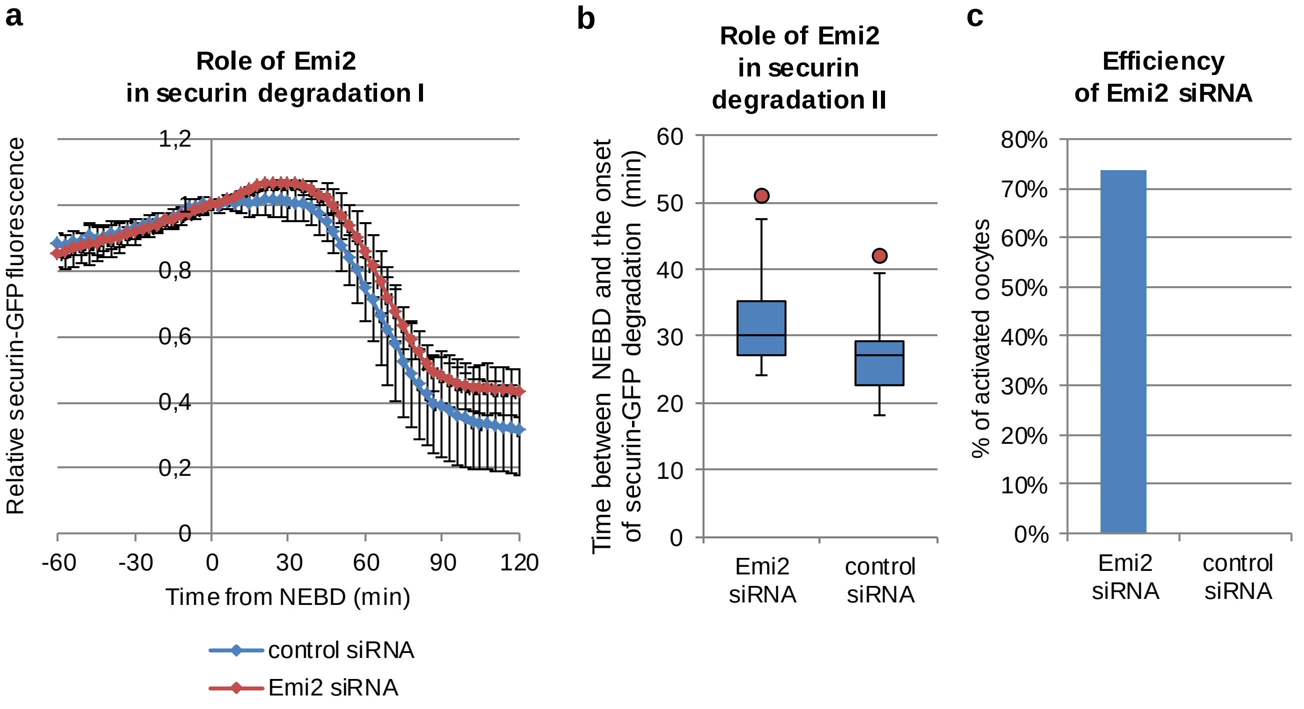

Supplementary Figure S3

siRNA-mediated depletion of Emi2 in oocytes and its impact on securin degradation

(**a**) Quantification of securin-GFP degradation in parthenogenotes injected with control and Emi2-specific siRNA, averaged from 6 and 18 embryos respectively. Plots show mean values +/- standard deviation. (**b**) Time between NEBD and the onset of securin-GFP degradation in parthenogenotes injected with control and Emi2-specific siRNA, averaged from 6 and 18 embryos respectively. The boxes show medians and the first and third quartiles. The whiskers are set at 1.5*IQR above the third and below the first quartile. Outlier values are marked with dots. (**c**) Percentage of oocytes activated after injection with control and Emi2-specific siRNA (n=8 and 34, respectively).

Supplementary Figure S4

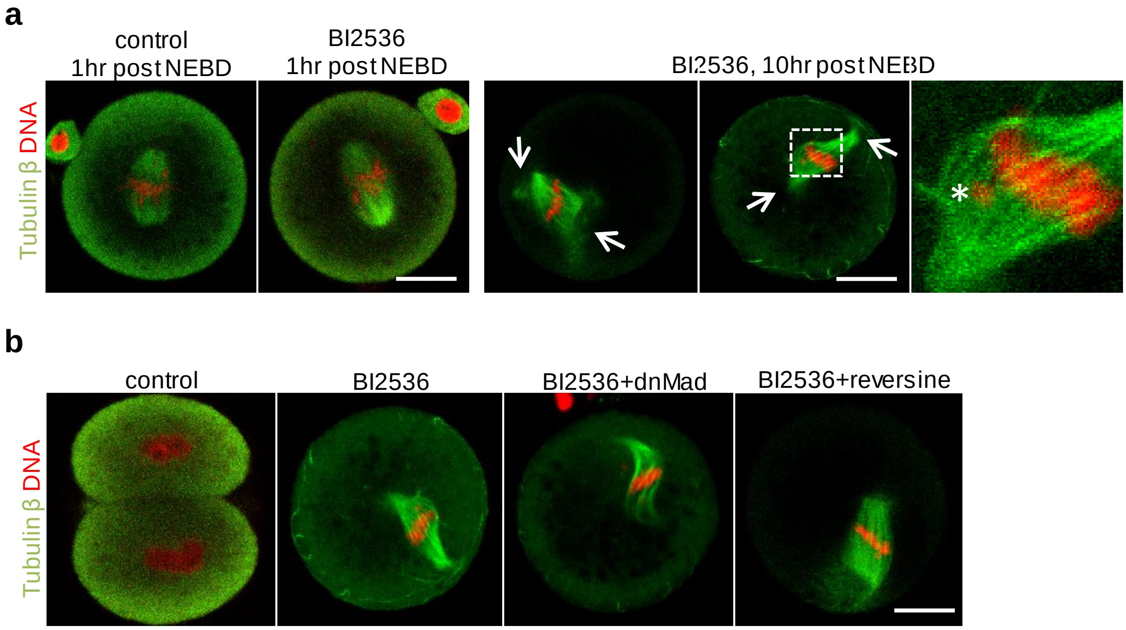

Supplementary Figure S4

Effect of Plk1 inhibition on mitotic spindle assembly and maintenance in zygotes

(**a**) Images of fixed zygotes stained for tubulin β (green) and chromatin (red). Arrows mark spindle poles, an asterisk – a chromosome dislocated from the metaphase plate, a dashed line surrounds a region enlarged in the far right image. (**b**) Effect of SAC inhibition on BI2536-induced M-phase block in zygotes. Images of fixed control embryos, embryos treated only with BI2536, injected with dn Mad2 and treated with BI2536, and treated with BI2536 and reversine stained for tubulin β (green) and chromatin (red). Scale bars 20 µm.

Supplementary Figure S5

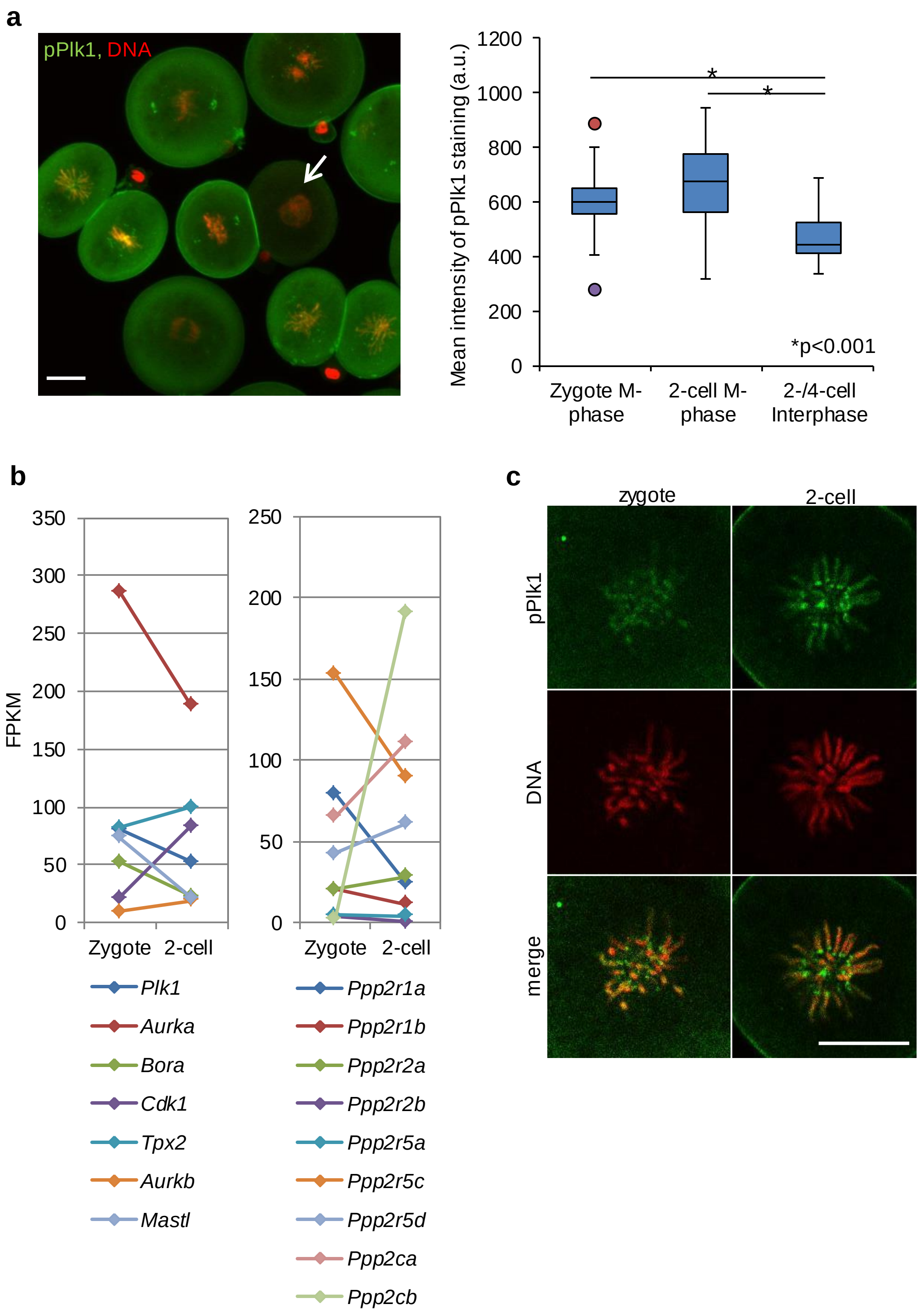

Supplementary Figure S5

Amount and distribution of phospho-Plk1 in embryos

(**a**) Levels of Plk1 phosphorylated at Thr210 (pPlk1) in M-phase zygotes and 2-cell embryos, and in 2- and 4-cell embryos in interphase. The image shows a representative z-stack projection of fixed embryos labeled for pPlk1 (green) and chromatin (red). Arrow indicates a 2-cell stage blastomere in interphase. Scale bar 20 μm. The graph shows quantification of the mean intensity of pPlk1 immunostaining. The boxes show medians and the first and third quartiles. The whiskers are set at 1.5*IQR above the third and below the first quartile. Outlier values are marked with dots. (**b**) Transcriptomic data (in fragments per kilobase million) regarding expression of Plk1 and its known activators (the left panel), and subunits of the protein phosphatase 2A (a Plk1 inhibitor; the right panel). Based on the DBTMEE database (RNA-seq Ver2_FPKM collection, http://dbtmee.hgc.jp/). (**c**) pPlk1 localization on M-phase chromosomes in zygotes and 2-cell embryos. Single-plane images of fixed embryos stained for pPlk1 (green) and chromatin (red). Scale bar 20 μm.
